# Supplementary material for: Awake Bruxism Identification: A Specialized Assessment Tool for Children and Adolescents—A Pilot Study
Source: Int J Environ Res Public Health. 2025 Jun 22;22(7):982. doi: 10.3390/ijerph22070982 (PMC12294309; doi:10.3390/ijerph22070982)
Supplement: Supplementary file 1 [file ijerph-22-00982-s001.zip › ijerph-3595131-supplementary.pdf]

Supplementary Material

This supplementary material contains:

(1) Awake Bruxism Identification Tool (ABIT) in Brazilian Portuguese

(2) Pilot Test of items the Tool in Brazilian Portuguese

(3) Awake Bruxism Identification Tool (ABIT) in English

(4) Pilot Test of items the Tool in English

Supplementary References

Data produced by the research

(1) Awake Bruxism Identification Tool (ABIT) in Brazilian Portuguese. Source: developed by research (PPGO - Faculdade de Odontologia de Piracicaba – Universidade Estadual de Campinas) - 5 pages

FICHA 1 – Ferramenta de Identificação do Bruxismo em Vigília

A ficha é composta por 5 itens e seu preenchimento é realizado em dois momentos distintos. Os itens 1 e 2 são preenchidos na entrevista inicial conduzida pelo pesquisador. Os itens 3, 4 e 5 são respondidos pelos participantes da pesquisa após o intervalo de 7 dias de observação.

*Nome da Criança:*___________________________________ *Nascimento:* ___________

Data da avaliação: ___/___/___

Folha 1 – Destinada ao Preenchimento pelo Pesquisador. Itens 1, 2 e 3

ITEM 1-RELATO DO RESPONSÁVEL (R1) - Entrevista realizada pelo pesquisador.

Sobre a criança/adolescente:

Você percebe que ele (a) **range** os dentes durante o dia (movimenta a mandíbula/queixo repetidamente para os lados e/ou para frente e para trás mantendo os dentes em contato/tocando um no outro?)<br><br>*Relato de ranger	Nunca	Quase nunca	Algumas vezes	Na maioria das vezes	Sempre	Não sei
Você percebe que ele (a) **aperta** os dentes durante o dia (aperta os dentes superiores contra os dentes inferiores, em um movimento de compressão/apertamento?)<br><br>*Relato de contato/apertamento dos dentes.	Nunca	Quase nunca	Algumas vezes	Na maioria das vezes	Sempre	Não sei
Você percebe que ele (a) apresenta rigidez/**contração da face** e/ou **queixo projetado**? (os músculos da face/rosto parecem "tensos-travados" ou o queixo é projetado/empurrado para frente ou para os lados?)<br><br>*Relato de reforço	Nunca	Quase nunca	Algumas vezes	Na maioria das vezes	Sempre	Não sei

0=nunca, 1=quase nunca, 2=algumas vezes, 3=na maioria das vezes e 4=sempre. Não sei=NS

* definição do tipo de comportamento em vigília (Lobbezoo et al., 2018):

ITEM 2 - AUTORRELATO (AR) – Entrevista realizada pelo pesquisador

A criança/adolescente responde:

Você **range** os dentes durante o dia (movimenta repetidamente a mandíbula/queixo de um lado para o outro e/ou para frente e para trás mantendo os dentes em contato/tocando um no outro?)<br><br>*Autorrelato de ranger	Nunca	Quase nunca	Algumas vezes	Na maioria das vezes	Sempre	Não sei
Você **aperta** os dentes durante o dia (aperta os dentes superiores contra os dentes inferiores, em um movimento de compressão /apertamento?)<br><br>*Autorrelato de contato/apertamento dos dentes.	Nunca	Quase nunca	Algumas vezes	Na maioria das vezes	Sempre	Não sei
Você apresenta rigidez/ **contração da face** e/ou **queixo projetado**? (os músculos da face/rosto parecem "tensos-travados" ou o queixo é projetado/empurrado para frente ou para os lados?)<br>*Autorrelato de reforço.	Nunca	Quase nunca	Algumas vezes	Na maioria das vezes	Sempre	Não sei

0 = nunca, 1 = quase nunca, 2 = algumas vezes, 3 = na maioria das vezes e 4 = sempre.

* definição do tipo de comportamento em vigília (Lobbezoo et al., 2018):

AUTORRELATO (AR)-AVALIAÇÃO ECOLÓGICA MOMENTÂNEA (AEM) LÚDICA Será preenchida por pesquisador após devolutiva da folha 4 pelo participante: pintura lúdica

Neste item o pesquisador irá anotar o resultado do item 4.

Pintou algum desenho durante observação de 7 dias?	SIM	NÃO
Se sim, em quantos dias são observdas uma ou mais pinturas?		
Quantas pinturas foram realizadas?		

ITEM 3 - RELATO DO RESPONSÁVEL (R2) - Destinada aos Pais/responsáveis

Senhor (a) responsável, o Sr. (a) está recebendo uma folha com perguntas, as quais deverão ser respondidas após 1 semana (7 dias), depois de observar o comportamento da criança/adolescente, conforme foi mostrado pela pesquisadora e exemplificada nas imagens/vídeo. Não existem respostas CERTAS ou ERRADAS. O importante é conhecermos o comportamento real da criança/adolescente. Para isso, pedimos que marque com um X apenas uma resposta a cada pergunta.

Responder após observação por 7 dias

Sobre a criança/adolescente:

Você percebeu se ele (a) **rangeu** os dentes durante o dia (movimentou a mandíbula/queixo repetidamente para os lados e/ou para frente e para trás mantendo os dentes em contato/tocando um no outro?)<br><br>*Relato de ranger	Nunca	Quase nunca	Algumas vezes	Na maioria das vezes	Sempre	Não sei
Você percebeu se ele (a) **apertou** os dentes durante o dia (apertou os dentes superiores contra os dentes inferiores, em um movimento de compressão/apertamento?)<br><br>*Relato de contato/apertamento dos dentes.	Nunca	Quase nunca	Algumas vezes	Na maioria das vezes	Sempre	Não sei
Você percebe que ele (a) apresentou rigidez/**contração da face** e/ou **queixo projetado**? (os músculos da face/rosto pareceram "tensos-travados" ou o queixo foi projetado/empurrado para frente ou para os lados?)<br><br>*Relato de reforço	Nunca	Quase nunca	Algumas vezes	Na maioria das vezes	Sempre	Não sei

0=nunca, 1=quase nunca, 2=algumas vezes, 3=na maioria das vezes e 4=sempre. Não sei=NS

* definição do tipo de comportamento em vigília (Lobbezoo et al., 2018):

Data da devolução:___/___/___

ITEM 4 – Destinada ao participante criança/adolescente da pesquisa

Caro participante da pesquisa, você está recebendo 1 folha com desenhos, a qual ficará com você por 7 dias. Conforme foi mostrado e exemplificado com imagens/ vídeo, você pode perceber que rangeu e apertou o dentes, ou que ficou com face contraída ou queixo projetado. Se um desse comportamentos ocorrer, pedimos que **pinte a carinha.**

PINTURA PARA 7 DIAS Data da devolução:___/___/___

Dia 1 – Dia da semana:_______________

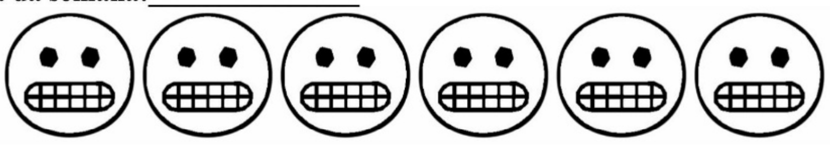

Dia 2 – Dia da semana:_______________

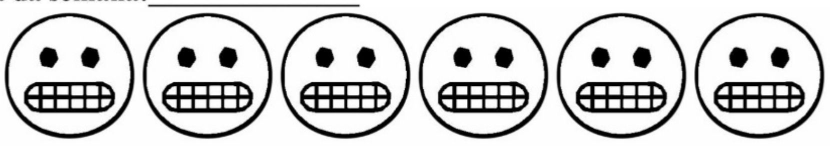

Dia 3 – Dia da semana:_______________

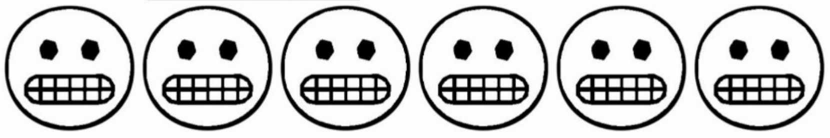

Dia 4 – Dia da semana:_______________

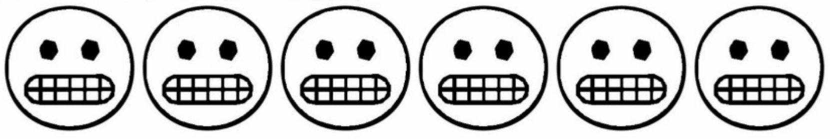

Dia 5 – Dia da semana:_______________

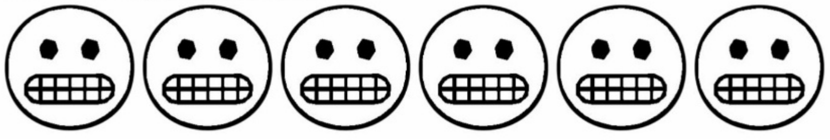

Dia 6 – Dia da semana:_______________

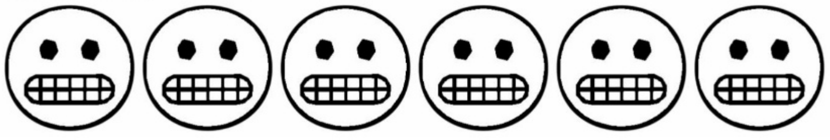

Dia 7 – Dia da semana:_______________

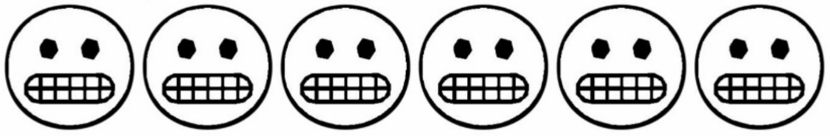

ITEM 5 - AVALIAÇÃO CLÍNICA - realizada pelo pesquisador

Exame clínico realizado com recursos de iluminação, secagem e espelho intrabucal. Pode ser realizada fora do ambiente odontológico.

1. EXAME EXTRAORAL

Hipertrofia de masseter?	E		D	
	S	N	S	N
Hipertrofia de temporal?	E		D	
	S	N	S	N
Presença de assimetria facial?	SIM		NÃO	
Dor na apalpação do músculo temporal?	SIM		NAO	
Dor na apalpação do músculo masseter?	SIM		NÃO	

2. EXAME INTRAORAL

**Edentações?**	Mucosa Labial	Língua	Mucosa Jugal
	Superior		Direita
	Inferior		Esquerda
**Hiperqueratose?**	Mucosa Labial	Língua	Mucosa Jugal
	Superior		Direita
	Inferior		Esquerda

Desgaste Dentário: Registrar o grau para cada elemento (Lobbezoo et al., 2001)

**17**	**16**	**15/55**	**14/54**	**13/53**	**12**	**11**		**21**	**22**	**23/63**	**24/54**	**25/55**	**26**	**27**

**47**	**46**	**85/45**	**84/44**	**83/43**	**42**	**41**		**31**	**32**	**33/73**	**34/74**	**35/75**	**36**	**37**

Grau 0: Dente hígido (sem perda da característica do esmalte);
Grau 1: Perda da característica da superfície do esmalte (desgaste em esmalte);
Grau 2: Desgaste visível com exposição de dentina e perda de altura clínica da coroa $\leq 1/3$;
Grau 3: Perda da altura da coroa $> 1/3$, mas menor que 2/3;
Grau 4: Perda de altura da coroa $\geq 2/3$

(2) Pilot Test of items the Tool in Brazilian Portuguese. Questions de understandability, feasibility and stability in Brazilian Portuguese. Source: developed by research (PPGO - Faculdade de Odontologia de Piracicaba – Universidade Estadual de Campinas) - 1 page

Pais/Responsáveis

COMPREENSÃO			
Você entendeu as perguntas da pesquisa realizada na entrevista?	Não Entendi Nenhuma	Entendi Parcialmente	Entendi Completamente
Você entendeu as perguntas da pesquisa realizada na sua casa?	Não Entendi Nenhuma	Entendi Parcialmente	Entendi Completamente
Qual sua dúvida?			

APLICABILIDADE / FACILIDADE / DIFICULDADE			
Qual o nível de dificuldade que a pesquisa criou na sua rotina?	Nenhuma	Baixa	Média
Qual sua sugestão para melhorar?			

TEMPO	Entrevista Inicial	Responder perguntas no Domicílio
Tempo destinado	Menos de 5 minutos	Menos de 5 minutos
	5 a 10 minutos	5 a 10 minutos
	Acima de 10 minutos	Acima de 10 minutos

Crianças

COMPREENSÃO			
Você entendeu as perguntas da pesquisa realizada na entrevista?	Não Entendi	Entendi Algumas partes	Entendi tudo
Você entendeu a tarefa de pintura para fazer em casa e na escola?	Não Entendi	Entendi Algumas partes	Entendi tudo
Qual sua dúvida?			

APLICABILIDADE / FACILIDADE / DIFICULDADE			
Qual foi dificuldade de participar da pesquisa na sua casa?	Nenhuma	Quase Nenhuma	Um pouco
Qual foi dificuldade de participar da pesquisa na escola?	Nenhuma	Quase Nenhuma	Um pouco
Qual sua sugestão para melhorar?			

TEMPO	Entrevista Inicial	Pintura dos Desenhos
Tempo destinado	Menos de 5 minutos	Menos de 5 minutos
	5 a 10 minutos	5 a 10 minutos
	Acima de 10 minutos	Acima de 10 minutos

(3) Awake Bruxism Identification Tool (ABIT) in Brazilian English. Source: developed by research (PPGO - Faculdade de Odontologia de Piracicaba – Universidade Estadual de Campinas) - 5 pages

FORM 1 – AWAKE BRUXISM IDENTIFICATION TOOL (ABIT)

The ABIT consists of 5 items and is completed in two separate stages. Items 1 and 2 are filled out during the initial interview conducted by the researcher. Items 3, 4, and 5 are answered by the research participants after a 7-day observation period. Item 6 is a clinical examination conducted by the researcher.

Child's Name: ________________________________ Date of Birth: ________________

Date of Assessment: _______________

ITEM 1 – REPORT FROM THE CAREGIVER – Interview conducted by the researcher

Do you notice if the child grinds their teeth?<br>Is the child's jaw repeatedly moved sideways and/or back and forth while the teeth re-main in contact?<br><br>*Questions for the awake child<br>*Report of teeth grinding	Never	Almost never	Sometimes	Most of the time	Always	I don't know
Did you notice the child clenching their teeth?<br>Are the upper teeth brought into contact with the lower teeth in a clenching motion?<br><br>*Questions for the awake child<br>*Report of teeth clenching	Never	Almost never	Sometimes	Most of the time	Always	I don't know
Do you notice the child bracing or thrusting the jaw?<br>Is it noticed whether the facial muscles appear tense or rigid (contracted held in a fixed position without contact with the teeth) or if the chin is held in a forward (protruded) or sideways (lateral) position?<br><br>*Questions for the awake child	Never	Almost never	Sometimes	Most of the time	Always	I don't know

0 = never, 1 = almost never, 2 = sometimes, 3 = most of the time, and 4 = always.
"definition of the type of wakefulness behavior"

ITEM 2 – SELF-REPORT – Interview conducted by the researcher

Do you grind your teeth?<br>Is your jaw repeatedly moved from side to side and/or back and forth while your teeth are kept in contact?)<br><br>*Questions for the awake child	Never	Almost never	Sometimes	Most of the time	Always	I don't know
Do you clench your teeth?<br><br>Is it noticed that your upper and lower teeth are clenched together?<br><br>*Questions for the awake child	Never	Almost never	Sometimes	Most of the time	Always	I don't know
Do you brace and/or thrust your jaw?<br>Is it noticed whether their facial muscles become tense or rigid (contracted), or if the chin is positioned forward (protruded) or to the side (lateral)?<br><br>*Questions for the awake child	Never	Almost never	Sometimes	Most of the time	Always	I don't know

0 = never, 1 = almost never, 2 = sometimes, 3 = most of the time, and 4 = always.
"definition of the type of wakefulness behavior"

EMA RESULT – To be completed by the researcher after the return of Sheet 3 by the participant: playful drawing

In this item, the researcher will record the result of the coloring activity from Item 5 completed by the research participant.

Did you color any drawings during the 7-day observation period?	YES	NO
How many drawings were colored?		
How many days show a record of coloring?		

ITEM 3 – To be completed by the caregiver

Dear caregiver

You are receiving a form with questions that should be answered after one week – 7 days – of observing your child's behavior, as demonstrated by the dentist. There are no RIGHT or WRONG answers. What matters is recognizing your child's actual behavior.

Please mark only one answer for each question with an X.

Questions for the awake child

Did you notice the child ground their teeth?<br><br>Was the child's jaw repeatedly moved sideways and/or back and forth while the teeth remained in contact?<br><br>*Questions for the awake child	Never	Almost never	Sometimes	Most of the time	Always	I don't know
Did you notice the child clenching their teeth?<br><br>Were the upper teeth brought into contact with the lower teeth in a clenching motion?<br><br>*Questions for the awake child	Never	Almost never	Sometimes	Most of the time	Always	I don't know
Do you notice the child bracing or thrusting their jaw?<br><br>Is it observed whether the facial muscles appear tense or rigid (contracted, held in a fixed position without contact with the teeth), or if the chin is held in a forward (protruded) or sideways (lateral) position?<br><br>*Questions for the awake child	Never	Almost never	Sometimes	Most of the time	Always	I don't know

0 = never, 1 = almost never, 2 = sometimes, 3 = most of the time, and 4 = always.
"definition of the type of wakefulness behavior"

Date of return:________/________/_________

ITEM 4 – EMA – TO BE COMPLETED BY THE RESEARCH PARTICIPANT

YOU ARE RECEIVING A SHEET WITH DRAWINGS.

YOU WILL KEEP IT WITH YOU FOR 7 DAYS.YOU SHOULD COLOR A “FACE” IF YOU NOTICE ANY BRUXISM BEHAVIOR.

THE BEHAVIORS ARE THOSE SHOWN AND EXPLAINED BY THE DENTIST. ONLY COLOR IF IT HAPPENS! IT’S OKAYIF YOU DON’T COLOR ANYTHING!

WHAT MATTERS IS TRUTHFUL INFORMATION.

PLAYFUL COLORING FOR 7 DAYS Date of return: ____________________

Day 1 – Day of the week: _______________

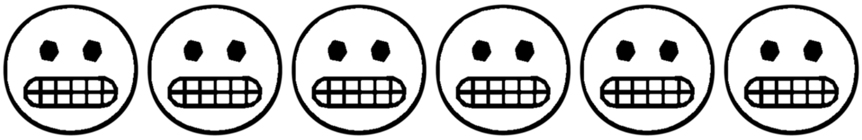

Day 2 – Day of the week: _______________

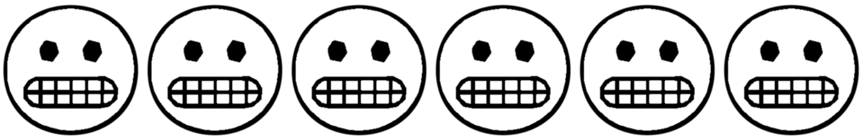

Day 3 – Day of the week: _______________

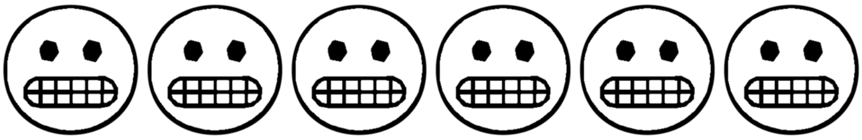

Day 4 – Day of the week: _______________

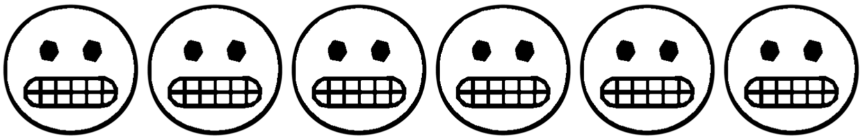

Day 5 – Day of the week: _______________

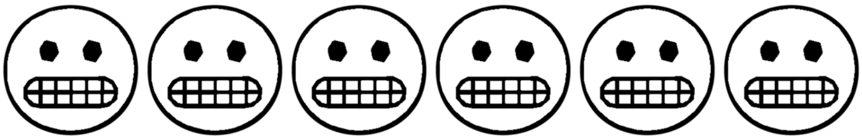

Day 6 – Day of the week: _______________

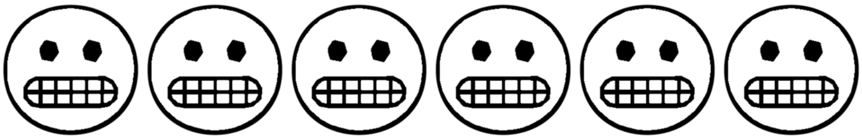

Day 7 – Day of the week: _______________

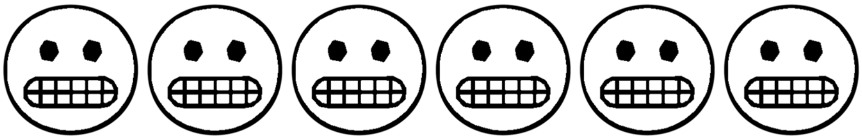

ITEM 5 – CLINICAL EXAMINATION FORM

The questions will be answered based on a clinical examination conducted by the researcher using lighting, drying, and dental materials. The examination may be performed outside the dental office environment.

EXTRAORAL EXAMINATION – Examination performed by the Dentist

Masseter hypertrophy?	**Right**		**Left**	
	YES	NO	YES	NO
Temporal hypertrophy?	**Right**		**Left**	
	YES	NO	YES	NO

Pain on palpation of the temporal muscle?	YES	NO
Pain on palpation of the masseter muscle?	YES	NO

Mark with an X if Yes:

Indentations?	**Labial Mucosa**	**Tongue**	**Buccal Mucosa**
	Upper		**Right**
	Lower		**Left**
Hyperkeratosis?	**Labial Mucosa**	**Tongue**	**Buccal Mucosa**
	Upper		**Right**
	Lower		**Left**

Tooth Wear: Record the degree for each tooth

17	16	15/55	14/54	13/53	12	11		21	22	23/63	24/54	25/55	26	27

47	46	85/45	84/44	83/43	42	41		31	32	33/73	34/74	35/75	36	37

Grade 0: Intact tooth (no loss of enamel surface characteristics)
Grade 1: Loss of enamel surface characteristics (wear limited to enamel)
Grade 2: Visible wear with dentin exposure and loss of clinical crown height ≤ 1/3
Grade 3: Loss of crown height > 1/3 but less than 2/3
Grade 4: Loss of crown height ≥ 2/3

(4) Pilot Test of items the Tool in English Questions de understandability, feasibility and stability in Brazilian Portuguese. Source: developed by research (PPGO - Faculdade de Odontologia de Piracicaba – Universidade Estadual de Campinas) - 1 page

Parent or Guardian

1. Did you understand the questions from the survey conducted during the interview?
( ) Did not understand any
( ) Partially understood
( ) Completely understood

2. Did you understand the questions from the survey conducted at your home?
( ) Did not understand any
( ) Partially understood
( ) Completely understood

3. What is your question or doubt?
Open response:

Parent or Guardian

1. What level of difficulty did the survey create in your routine?
( ) None
( ) Low
( ) Medium
( ) High

2. What is your suggestion for improvement?
Open response:

Child

1. Did you understand the questions from the survey conducted during the interview?
( ) Did not understand any
( ) Understood some parts
( ) Understood everything

2. Did you understand the painting task to do at home and at school?
( ) Did not understand any
( ) Understood some parts
( ) Understood everything

3. What is your question or doubt?
Open response:

Child

1. What was the difficulty of participating in the survey at your home?
( ) None

( ) Almost none
( ) A little
( ) A lot

2. What was the difficulty of participating in the survey at school?
( ) None
( ) Almost none
( ) A little
( ) A lot

3. What is your suggestion for improvement?
Open response:

TIME

Time allocated
( ) Less than 5 minutes
( ) 5 to 10 minutes
( ) More than 10 minutes

Initial Interview
( ) Less than 5 minutes
( ) 5 to 10 minutes
( ) More than 10 minutes

Answering at Home
( ) Less than 5 minutes
( ) 5 to 10 minutes
( ) More than 10 minutes

Drawing Painting
( ) Less than 5 minutes
( ) 5 to 10 minutes
( ) More than 10 minutes

Record Changes in the Child's Records______________________________________

Record Changes in the Adult's Records______________________________________
